# Replication of linkage at chromosome 20p13 and identification of suggestive sex-differential risk loci for autism spectrum disorder

**DOI:** 10.1186/2040-2392-5-13

**Published:** 2014-02-17

**Authors:** Donna M Werling, Jennifer K Lowe, Rui Luo, Rita M Cantor, Daniel H Geschwind

**Affiliations:** 1 Interdepartmental PhD Program in Neuroscience, Brain Research Institute, University of California, Los Angeles, 90095 Los Angeles, CA, USA; 2 Neurogenetics Program and Department of Neurology, David Geffen School of Medicine, University of California, Los Angeles, 90095 Los Angeles, CA, USA; 3 Center for Autism Research and Treatment, Semel Institute, David Geffen School of Medicine, University of California, Los Angeles, 90095 Los Angeles, CA, USA; 4 Center for Neurobehavioral Genetics, Semel Institute, University of California, Los Angeles, 90095 Los Angeles, CA, USA; 5 Department of Human Genetics, University of California, Los Angeles, 695 Charles E. Young Dr. South, Gonda 2506, 90095 Los Angeles, CA, USA

**Keywords:** Male brain, Sex differences, Intermediate phenotype, Linkage analysis, Association, AGRE

## Abstract

**Background:**

Autism spectrum disorders (ASDs) are male-biased and genetically heterogeneous. While sequencing of sporadic cases has identified *de novo* risk variants, the heritable genetic contribution and mechanisms driving the male bias are less understood. Here, we aimed to identify familial and sex-differential risk loci in the largest available, uniformly ascertained, densely genotyped sample of multiplex ASD families from the Autism Genetics Resource Exchange (AGRE), and to compare results with earlier findings from AGRE.

**Methods:**

From a total sample of 1,008 multiplex families, we performed genome-wide, non-parametric linkage analysis in a discovery sample of 847 families, and separately on subsets of families with only male, affected children (male-only, MO) or with at least one female, affected child (female-containing, FC). Loci showing evidence for suggestive linkage (logarithm of odds ≥2.2) in this discovery sample, or in previous AGRE samples, were re-evaluated in an extension study utilizing all 1,008 available families. For regions with genome-wide significant linkage signal in the discovery stage, those families not included in the corresponding discovery sample were then evaluated for independent replication of linkage. Association testing of common single nucleotide polymorphisms (SNPs) was also performed within suggestive linkage regions.

**Results:**

We observed an independent replication of previously observed linkage at chromosome 20p13 (*P* < 0.01), while loci at 6q27 and 8q13.2 showed suggestive linkage in our extended sample. Suggestive sex-differential linkage was observed at 1p31.3 (MO), 8p21.2 (FC), and 8p12 (FC) in our discovery sample, and the MO signal at 1p31.3 was supported in our expanded sample. No sex-differential signals met replication criteria, and no common SNPs were significantly associated with ASD within any identified linkage regions.

**Conclusions:**

With few exceptions, analyses of subsets of families from the AGRE cohort identify different risk loci, consistent with extreme locus heterogeneity in ASD. Large samples appear to yield more consistent results, and sex-stratified analyses facilitate the identification of sex-differential risk loci, suggesting that linkage analyses in large cohorts are useful for identifying heritable risk loci. Additional work, such as targeted re-sequencing, is needed to identify the specific variants within these loci that are responsible for increasing ASD risk.

## Background

Autism spectrum disorders (ASDs) are a group of neurodevelopmental conditions characterized by severe social impairment that affect 1 in 88 individuals [1]. Genetic factors have long been known to contribute significantly to ASD risk based on twin studies [2], the sibling recurrence risk [3,4], and elevated rates of comorbid ASD in populations with a wide variety of monogenic syndromes such as Fragile X or Timothy Syndrome [5,6]. ASDs are also known to present heterogeneously across the population of affected individuals, and results from recent genetic studies strongly suggest that genetic risk factors for ASD are similarly diverse. Copy number variant (CNV) and exome sequencing studies of sporadic ASD cases from single-incidence (“simplex”) families have found numerous novel, *de novo* risk variants [7-13], and no significant signal for rare inherited variation. Estimates based on these findings project that approximately 1,000 genes are likely to contribute to ASD etiology.

While a highly productive approach for gene discovery, the study of simplex families is designed to identify mostly the non-inherited genetic component of ASD risk: rare variants resulting from *de novo* mutations, in which variants arise in the germ cell and are not carried by the mother or father. However, evidence of high heritability for ASD [14], high sibling recurrence risk [3,4], and aggregation of subthreshold ASD-like phenotypes in families [15-18] suggest that inherited genetic variation also plays a significant role in ASD etiology.

Additionally, while germline mutations, potentially shared between affected siblings, may also plausibly affect ASD risk in multiplex families, current evidence suggests that rare *de novo* CNV events are more prevalent among sporadic cases than cases from multiple-incidence (“multiplex”) families [13]. Largely, however, the specifics of the genetic architecture of ASDs that differ between simplex and multiplex families are unknown. Therefore, studies of familial transmission to identify regions of genetic linkage in multiplex families remain an important approach to identifying predisposing genes.

Another important clue to ASD etiology lies in its consistently male-biased prevalence [19]. There is an approximately 4:1 male bias, a phenomenon that is likely driven, or at least influenced, by the actions of sex-specific biological factors, such as sex chromosomes or steroid hormones that potentiate and attenuate ASD risk in males and females, respectively [20]. Indeed, several ASD and intellectual disability risk genes have been identified on the X chromosome [6,21], including *FMR1*[22], *NLGN4X*, and *NLGN3*[23], demonstrating that in some cases ASD may be X-linked. However, the proportion of ASD cases currently attributable to X-linked variants remains insufficient to account for the degree of male bias observed in ASD prevalence. The notion of female protective factors on a broader scale is supported by the observation of an increased proportion of autistic females relative to males carrying variants of large effect size, such as large CNVs or deleterious single nucleotide variants (SNVs) [8,11,13]. However, with few exceptions [24], it is unknown which specific autosomal risk variants are differentially penetrant by sex, thus contributing to the sex bias in ASD prevalence. For example, a greater number of variants may be associated with ASD risk in males as compared to the number of variants that also, or specifically, confer risk to females. The discovery of such sex-differential risk loci would provide genetic clues for investigation of the biological mechanisms driving the ASD male bias. However, because these signals are likely masked by heterogeneity within sex-mixed cohorts, stratification of multiplex ASD family cohorts by proband sex may facilitate the identification of novel, sex-differential loci harboring inherited risk variants.

Most previous linkage studies of ASD have used relatively small samples (<350 families) and markers with coarse resolution and incomplete information [25-31], and there has been little agreement between studies in the reported findings. Furthermore, the larger studies [32,33] combine subjects drawn from several diverse populations with different ascertainment schemes. Multiplex family samples ranging from 109 to 753 families from the Autism Genetics Resource Exchange (AGRE) cohort have been previously tested for linkage [25-28,33,34], and the linked risk loci reported by these analyses, with a few exceptions [25,28], show little agreement. This may be due to genetic heterogeneity, small sample sizes, or sparse marker coverage.

**Figure 1:**
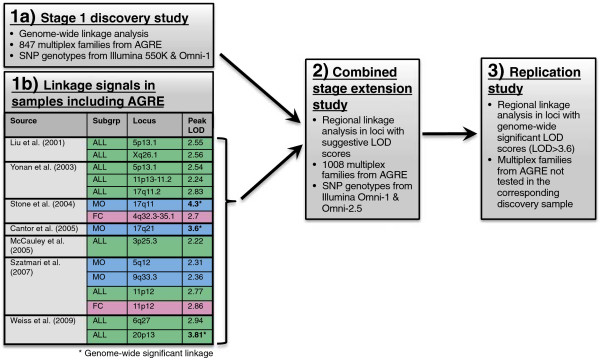
**Schematic of three-stage study design for linkage analyses.** Linkage analyses were performed using a three-stage approach. **(1a)** A discovery sample (Stage 1) and **(1b)** previous linkage results were used to identify loci of suggestive linkage (logarithm of odds (LOD) ≥2.2). **(2)** These suggestive loci were tested for linkage in an extended sample. **(3)** For loci achieving genome-wide significant linkage (LOD ≥3.6) in either the discovery, extended, or previously published studies, the non-overlapping subset of families was tested for independent replication of linkage. AGRE, Autism Genetics Resource Exchange; SNP, single nucleotide polymorphism.

In this study, we used a pruned set of single nucleotide polymorphism (SNP) markers providing nearly complete linkage information on all autosomes and the X chromosome (information content greater than 0.976 for 99.5% of regions covered by SNP genotypes) for our analyses in the largest available AGRE sample. Specifically, we performed non-parametric linkage testing in three stages to: 1) identify novel risk loci in a discovery sample of 847 multiplex families; 2) confirm loci identified in this discovery sample, or reported by earlier analyses of AGRE samples, in an extended sample of 1,008 multiplex families; and 3) test for formal replication of genome-wide significant linkage signals in the independent portion of families not tested in the corresponding discovery studies (Figure 1).

For all stages, we also applied a stratification approach used previously [25,28,32] to identify sex-differential loci, now using a sample three times as large as the original and a panel of markers that are substantially more informative [28]. We then used the family-based association transmission disequilibrium test (TDT) on genotyped and imputed SNPs within linked regions to identify common variants conferring increased risk for ASD. With the addition of 343 AGRE families beyond those tested in the most recent linkage analysis using subjects from AGRE [33], this comprises the largest linkage study for ASD, and sex-differential risk, of families from a single, uniformly ascertained cohort. This also allows us to attempt replication of previous linkage findings.

## Methods

### Subjects and genotyping

Subjects were individuals from the AGRE collection of nuclear families, including affected probands, their unaffected siblings, and their parents. Subjects provided written informed consent or, if minors, assent with agreement from their parents to AGRE for diagnosis and blood collection. This study was approved by the Western Institutional Review Board (AGRE), the Institutional Review Board at Washington University (subject recruitment, principal investigator: John Constantino), and by the Medical Institutional Review Board 3 at the University of California, Los Angeles.

Individuals with study diagnoses of full autism, not quite autism, or broad spectrum disorder based on a clinician’s best estimate given Autism Diagnostic Interview-Revised and Autism Diagnosis Observation Schedule scores were considered to be affected, in accordance with the inclusive, single spectrum concept of ASD as now codified in the Diagnostic and Statistical Manual-5. In the AGRE cohort, a study diagnosis of not quite autism is given to subjects who meet the age of onset criterion but score no more than one point short of the autism cutoffs in one or more symptom domains (social, communication, restricted and repetitive behavior), or subjects who meet autism cutoffs in all symptom domains, but do not meet the age of onset criterion. A study diagnosis of broad spectrum disorder is given to subjects with pervasive developmental disorders and varying levels of impairment; these subjects include individuals with conditions like pervasive developmental disorder not otherwise specified (PDD-NOS) and Asperger’s syndrome.

Probands with syndromic autism, significant dysmorphology, documented pre- or peri-natal insult, abnormal imaging or medical test, premature birth at less than 35 gestational weeks, or chromosomal abnormalities were not included; additional neuropsychiatric phenotypes in parents and unaffected siblings were not applied as inclusion or exclusion criteria. Here, chromosomal abnormalities refer to clinically relevant CNVs identified by karyotype and to recurrent CNVs such as 16p11.2 deletions and duplications independently reported to AGRE by investigators. Subjects with evidence for *de novo* missense SNVs (n = 5 cases) [9] were not excluded, due to the current uncertainty in determining the effects of missense variation on ASD risk. In instances of monozygotic multiples, only one proband was selected at random for inclusion. Subjects in the AGRE cohort include individuals of Caucasian, African-American, Asian, and Hispanic ancestry as noted by self-report and multi-dimensional scaling from genotype data; subjects were not filtered by ancestry, as the genetic analyses used in this study (non-parametric linkage, TDT) were family-based and therefore not susceptible to the introduction of false positive results from population stratification. However, we note that including multiple ethnicities may introduce or exacerbate locus heterogeneity, which is unlikely to falsely inflate logarithm of odds (LOD) scores, but instead may reduce power in linkage studies [32].

Subjects were genotyped in two stages, using DNA purified from lymphoblastoid cell lines and obtained from the Rutgers University Cell and DNA Repository (Piscataway, NJ, USA). Stage 1 consisted of individuals from 1,191 AGRE families, with subjects from 941 families genotyped on the Illumina 550K genome-wide SNP array as described previously [35], and individuals from an additional 250 AGRE families typed on the Illumina Omni-1 Quad array (Illumina, San Diego, CA, USA) in the University of California, Los Angeles, Neuroscience Genomics Core. Stage 2 consisted of individuals from an additional 396 AGRE families, 116 of which were genotyped on the Illumina Omni-1 array, and 280 of which were typed on the Illumina Omni-2.5 array (Additional file 1: Table S1). In total, the combined sample of stage 1 plus stage 2 was comprised of individuals from 1,587 families, 1,008 of which were multiplex and met inclusion criteria as described above.

Recorded sample identity and pedigree relationships were validated by evaluating estimations of identical by descent allele sharing across the genome within and between families using PLINK software [36]. In cases with evidence of identity swaps, where available, genotype data from the Broad Institute (Affymetrix 5.0 [33]) and the Autism Genome Project (Illumina 1M [37,38]) were compared to make a final determination of identity. Subjects and SNPs with >5% missing data were excluded, and SNPs with Hardy-Weinberg equilibrium *P*-values <0.0000001, minor allele frequency <0.01, and >10 Mendelian errors were also excluded. Filtered data sets from the two stage 1 genotyping platforms were then merged using PLINK to generate a stage 1 sample data set with all remaining subjects and the union of marker sets from the 550K and Omni-1 platforms for a total of 1,092,577 SNPs. To incorporate stage 2 and build the combined sample data set of all genotyped AGRE subjects, the union of filtered data from all genotyping platforms (550K, Omni-1, Omni-2.5) were merged using PLINK for a total of 1,684,432 SNPs.

### Linkage analyses using all families

Autosomal and X chromosome markers common to all platforms and pruned to a linkage disequilibrium r^2^ ≤ 0.1 with PLINK were used for genome-wide linkage analysis (stage 1 data: 57,929 SNPs; combined stage data: 53,648 SNPs). These sets of independent SNPs were mapped to genetic positions using Rutgers Combined Linkage-Physical maps [39,40] for linkage testing. Non-parametric, multipoint linkage was performed genome-wide on all stage 1 multiplex families meeting inclusion criteria (n = 847 families) using Merlin (autosomes) and Minx (X chromosome, executable option within Merlin) [41], which applies the Kong and Cox linear model [42] to test for small increases in allele sharing across a large sample of families.

Linked regions identified in the stage 1 sample or by previous reports (discovery samples) from non-parametric linkage analyses of AGRE samples using the “broad” affection status criteria were then identified for further evaluation by defining the 2-LOD interval surrounding suggestive (LOD ≥2.2) and significant (LOD ≥3.6) signals. Since the precise physical position of linkage peaks may vary between studies using marker sets of differing density and information content (see Cantor and colleagues [25] for an example), this 2-LOD interval is intended to be inclusive and to encompass any underlying variability in the location of previous signals. Where there were discrepancies between the genetic maps from the original studies and our data, the span of the interval was anchored on the current physical position (hg19) of the peak marker. Linkage signals from the study by Szatmari and colleagues [32] were reported as Z_|r_ scores, which we converted to LOD scores by: LOD = Z_|r_^2^/(2*ln(10)) [42]. Regions with suggestive linkage evidence from stage 1 or previous reports were tested for linkage in the combined sample. Regions at which the discovery sample showed genome-wide significant linkage (LOD ≥3.6) were then evaluated for replication (*P* < 0.01) within a 2-LOD interval from the peak LOD by testing only those independent families not previously evaluated in the corresponding discovery analysis.

We applied this three-step (1, discovery; 2, extension and 3, replication) approach according to guidelines for linkage analyses of complex traits outlined by Lander and Kruglyak [43]. As preliminary linkage analyses for complex traits have yielded suggestive signals, extending sample sizes by adding pedigrees may allow these loci to reach genome-wide significance. Once a genome-wide significant signal has been identified, it may then be tested for independent replication in a new sample. The platform and stage at which a family was genotyped determined whether they were included in the discovery (stage 1) or the extension (combined stage) stages (Figure 1).

### Sex-stratified linkage

To identify sex-differential ASD risk loci, we stratified the genotyped, multiplex families into two groups based on the sex of their affected children: male-only (MO, no affected daughters), and female-containing (FC, at least one affected daughter) [28]; the number of affected-female-only multiplex families enrolled in AGRE is currently too low to analyze these families as their own subgroup. In keeping with earlier sex-stratified linkage analyses of AGRE families [25,28,32] and to maintain workable sample sizes, all families with two or more affected members were assigned by this simple criterion to either the MO or FC subset, irrespective of the total affected family members or the presence of unaffected siblings of the opposite sex. While it can be argued that MO families with three or more affected brothers or with at least one unaffected sister are more likely to carry truly male-specific risk variants (not penetrant in females) than MO families with fewer affected brothers or no unaffected sisters, the number of available AGRE families fitting these criteria is quite low. Specifically, 52 MO families include three or more affected brothers, and 150 MO families have recorded unaffected sisters. To avoid further restricting the sample size of our subsets, we define the MO and FC subsets simply by the sex of the two or more affected siblings, under the assumption that the MO and FC subsets are enriched for male-specific and female-affecting risk variants, respectively, as compared with the full, non-stratified sample. By this definition, the stage 1 sample consisted of 487 MO (61%) and 314 FC (39%) families, and the combined sample consisted of 602 MO (60%) and 406 FC (40%) families (Additional file 1: Table S1).

Linkage analyses were performed separately in the two subgroups in three steps as described above for the non-stratified sample: 1) genome-wide non-parametric linkage analysis of MO and FC subgroups from the stage 1 sample; 2) regional linkage analysis within suggestive (LOD ≥2.2) MO- or FC-specific linkage peaks from discovery studies [25,28] using the corresponding MO or FC subgroup from the combined sample; and 3) regional linkage analysis within peaks of genome-wide significant LOD (≥3.6) in the independent portion of the corresponding MO or FC subgroup from the combined sample who were not previously tested in the discovery study.

Additionally, to assess the statistical significance of sex-differential linkage signals, a randomization test of 10,000 subsets of 487 families (matching stage 1 MO family N) and 314 (stage 1 FC family N) were analyzed for linkage across chromosomes 1, 4, 6, and 8, where subgroup-specific suggestive linkage peaks (LOD ≥2.2) were observed. For the combined sample, random subsets of 602 (MO family N) were analyzed across chromosome 1, the only chromosome on which a sex-differential signal surpassed LOD 2.2 in the combined sample. On a marker-by-marker basis, the LOD score from each of the 10,000 random trials was compared to the results from the corresponding original, subset-specific scan, and the fraction of random trials for which LOD_random_ > LOD_original_ was taken as the empirical *P*-value for stratification, reflecting the frequency with which the observed LOD magnitude would occur under the null hypothesis of no linkage.

### Imputation

To further improve genotype coverage within linked regions for fine-scale association testing, imputation was performed separately by data set, as defined by genotyping platform and data collection stage, using IMPUTE2 [44] and a cosmopolitan reference panel from the 1000 Genomes Project [45]. Imputed SNPs from each data set were then merged using GTOOL [46], and SNPTEST [46] was used to generate summary statistics for the merged set. Data were filtered to SNPs with an IMPUTE2 quality score ≥0.5, missing data in ≤5% of subjects, minor allele frequency ≥1%, and Hardy Weinberg *P* ≥ 0.0000001. The final data set included 5,814,564 autosomal SNPs.

### Linkage-directed association testing

Imputed SNPs within the 2-LOD intervals surrounding linkage peaks exceeding the suggestive threshold (LOD ≥2.2) in either the stage 1 or combined sample were tested for association with ASD affection status in the family group corresponding to the linkage peak (all families (ALL), MO, or FC) using a TDT with adaptive gene-dropping permutations (PLINK). Gene dropping assignments are applied consistently across siblings to control for linkage and appropriately treat trios from multiplex families as non-independent [36].

In regions of MO or FC linkage signal, extended families without multiple affected siblings, but instead with multiple affected cousins, were additionally included in the association analysis (total MO n = 606, including 4 additional extended families; total FC n = 407, including 1 additional extended family). In regions of linkage signal from ALL, an additional 5 extended families and 508 families with only one genotyped, affected individual were included in the association analysis (total ALL n = 1,521 families). All additional families met inclusion criteria as described previously. Association *P*-values were adjusted for multiple testing according to the number of independent SNPs within each region, as defined by a pairwise linkage disequilibrium r^2^ < 0.3 (PLINK).

## Results

By testing the largest available, uniformly ascertained ASD family sample (n = 1,008 multiplex families) for linkage, we aimed to identify novel ASD risk loci not identified by the previous, smaller analyses of AGRE samples, and/or to confirm the loci reported by these analyses. As genotype data were collected in two stages, we use the stage 1 sample to identify linked and sex-differential risk loci genome-wide, and the combined sample (union of stages 1 and 2) to evaluate identified loci from stage 1 and earlier reports (discovery studies) for confirmation of linkage in the largest available AGRE genotype data set.

### Linkage in all families

**Figure 2:**
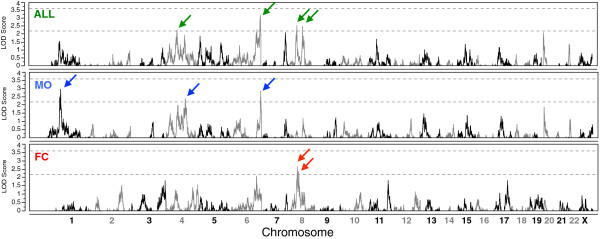
**Genome-wide linkage: stage 1 sample.** Logarithm of odds (LOD) scores from genome-wide, non-parametric, multipoint linkage analysis for autism spectrum disorder affection status in the stage 1 sample are plotted for autosomes (Merlin) and chromosome X (Minx). Top panel: all multiplex families (ALL); middle: male-only families (MO); bottom: female-containing families (FC). Dashed lines mark LOD thresholds 2.2 for suggestive and 3.6 for significant linkage [39]. Arrows note signals with LOD ≥2.2.

Non-parametric, genome-wide linkage analysis for ASD affection status in all stage 1 multiplex families (n = 847) identified four genomic regions with a peak LOD score ≥2.2 (Table 1, Figure 2), the threshold for suggestive linkage for a complex trait when allele sharing is tested in sibling pairs [43]. We observed the highest LOD score at chromosome 6q27, with LOD_ALL.St1_ = 3.22 at rs4708676 (190.611 cM). A 2-LOD interval from this peak SNP spans 18.2 Mb, 31.6 cM, and 100 RefSeq genes (Additional file 2: Figure S1A), and the peak SNP is 75 kb upstream from gene *FRMD1* [GenBank:NM_024919] (0.4-LOD drop from peak), a gene with a role in immune function and significantly associated with IL-2 secretion [47]. All other linkage regions yielded peak LOD ≥2.2 and are located at chromosomes 4q13.1 (LOD_ALL.St1_ = 2.3), 8p21.2 (LOD_ALL.St1_ = 2.55), and 8q13.2 (LOD_ALL.St1_ = 2.5).

**Table 1:** Summary of significant and suggestive linkage peaks from AGRE samples

**Discovery sample**					**Combined sample (Stage 1 + Stage 2)**			
**Source**	**Number of families**	**Subgroup**	Locus*	**Peak LOD**	**Number of families**	**Peak LOD**	**Peak *P *-value**	**Peak SNP**
Liu *et al.* (2001) [26]	110	ALL	5p13.1	2.55	1008	0.99	0.0163	rs6884342
	110	ALL	Xq26.1	2.56	1008	1.12	0.0116	rs12557711
Yonan *et al.* (2003) [34]	345	ALL	5p13.1	2.54	1008	0.99	0.0163	rs6884342
	345	ALL	11p13-11.2	2.24	1008	0.92	0.0199	rs2984699
	345	ALL	17q11.2	2.83	1008	0.53	0.0592	rs1382779
Stone *et al.* (2004) [28]	148	MO	17q11	**4.3**	602	0.42	0.0834	rs4795708
	109	FC	4q32.3-35.1	2.7	406	1.10	0.0121	rs1717072
Cantor *et al.* (2005) [25]	196	MO	17q21	**3.6**	602	0.47	0.0706	rs1877032
McCauley *et al.* (2005) [27]	158 (85 AGRE)	ALL	3p25.3	2.22	1008	0.04	0.3344	rs1400207
Szatmari *et al.* (2007) [32]	741 (211 AGRE)	MO	5q12	2.31	602	1.42	5.34e-3	rs706725
	741 (211 AGRE)	MO	9q33.3	2.36	602	1.47	4.64e-3	rs204169
	1181 (387 AGRE)	ALL	11p12	2.77	1008	0.92	0.0199	rs2984699
	440 (176 AGRE)	FC	11p12	2.86	406	0.38	0.0925	rs404977
Weiss *et al.* (2009) [33]	904 (753 AGRE)	ALL	6q27	2.94	1008	2.50^†^	3.43e-4	rs6931082
	904 (753 AGRE)	ALL	20p13	**3.81**	1008	3.02^†^	9.55e-5	rs6139007
Current study Stage 1 sample	487	MO	1p31.3	2.98	602	2.55^†^	3.05e-4	rs7521242
	847	ALL	4q13.1	2.30	1008	2.14	8.37e-4	rs1483288
	487	MO	4q26	2.41	602	1.28	7.60e-3	rs2196712
	847	ALL	6q27	3.22	1008	2.50^†^	3.43e-4	rs6931082
	487	MO	6q27	2.86	602	2.07	1.02e-3	rs960145
	314	FC	8p21.2	2.67	406	1.42	5.25e-3	rs7001120
	847	ALL	8p21.2	2.55	1008	2.18	7.60e-4	rs13257637
	314	FC	8p12	2.37	406	1.34	6.51e-3	rs2976525
	847	ALL	8q13.2	2.50	1008	2.82^†^	1.58e-4	rs4738003

*Refer to Additional file 3: Table S2 for locus details, including boundaries and peak markers.

**Bold type** indicates logarithm of odds (LOD) scores passing genome-wide significance thresholds of LOD ≥3.6 for discovery studies,

^†^LOD scores passing suggestive linkage thresholds of LOD ≥2.2 in the combined sample extension study. AGRE, Autism Genetics Resource Exchange; ALL, all families; FC, female-containing; MO, male-only; SNP, single nucleotide polymorphism.

**Figure 3:**
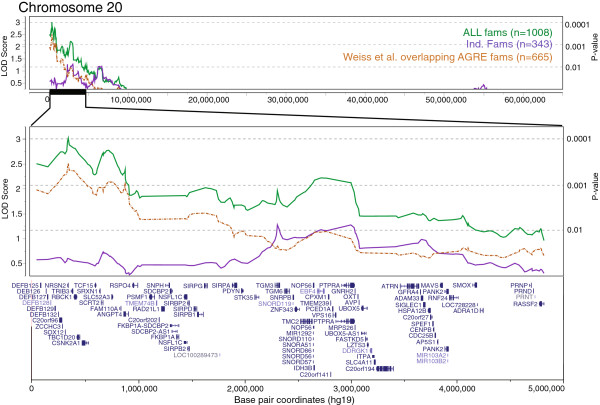
**Independent replication of genome-wide significant linkage at 20p13.** Genome-wide significant linkage signal at 20p13 from the combined sample (ALL fams; green, solid line), Autism Genetics Resource Exchange families (AGRE fams) analyzed by Weiss *et al.*[33] (orange, dashed line), and all AGRE families not previously analyzed by Weiss *et al. *[33] (Ind. Fams; purple, solid line). Top: linkage across the full chromosome 20; middle: linkage across a 2-logarithm of odds (LOD) interval from the peak LOD; bottom: RefSeq gene alignment in the 2-LOD interval. Dashed lines mark LOD thresholds corresponding to linkage *P*-values of 0.01, 0.001, and 0.0001 [35].

**Figure 4:**
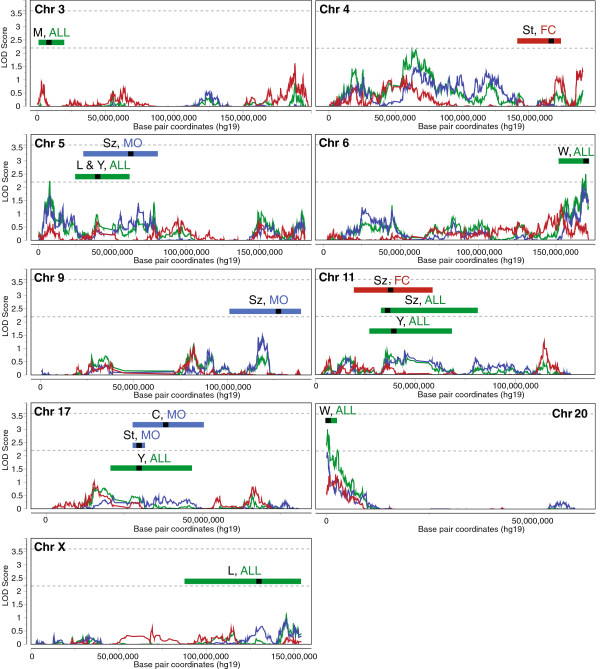
**Linkage in regions of interest from previous studies: combined sample.** Logarithm of odds (LOD) scores from non-parametric, multipoint linkage analysis for autism spectrum disorder affection status in the combined sample (Merlin) are plotted for chromosomes with suggestive linkage peaks (LOD ≥2.2) from previous studies. Colored bars and black marks indicate the spans and peaks of linkage regions of interest, respectively; text indicates the linkage region source (L, Liu *et al.*, 2001 [26]; Y, Yonan *et al.*, 2003 [34]; St, Stone *et al.*, 2004 [28]; C, Cantor *et al.*, 2005 [25]; M, McCauley *et al.*, 2005 [27]; Sz, Szatmari *et al.*, 2007 [32]; W, Weiss *et al.*, 2009 [33]; green and ALL, all multiplex families; blue and MO, male-only families; red and FC, female-containing families). Dashed lines mark LOD thresholds of 2.2 for suggestive and 3.6 for significant linkage.

To determine if the signals observed in the stage 1 sample and in previous AGRE studies could be improved or replicated in a larger sample size, we next carried out an extension study by testing these regions for linkage in the combined sample of 1,008 multiplex families (union of stage 1 and stage 2 samples). Non-parametric linkage analysis of the combined sample within the regions of interest confirmed three loci above the suggestive threshold of LOD ≥2.2: 6q27 (LOD_ALL.Com_ = 2.50; Additional file 2: Figure S1A), 8q13.2 (LOD_ALL.Com_ = 2.82; Additional file 2: Figure S1B), and 20p13 (LOD_ALL.Com_ = 3.02; Figure 3). Of these three loci, only the signal at 8q13.2 increased in the combined sample. Combined sample linkage on other chromosomes of interest is shown in Figure 4.

The strongest signal that we observed from the combined sample, and the only signal that surpassed a LOD score of 3.0, is at 20p13. This locus had been identified previously in a study that combined 753 AGRE families with other families from the National Institute of Mental Health repository [33]. Since 665 of the families in that analysis overlapped with the current sample, we formally tested for replication of this locus by analyzing only those families unique to our study (n = 343 multiplex families) [33]. We identified a linkage peak within the 20p13 region of interest with a *P*-value of 0.0076 at rs214828, thus meeting the significance threshold for independent replication of linkage for a complex trait (*P* < 0.01) [43]. An additional five nearby SNPs also had *P*-values of less than 0.01 in this independent family set. From the peak LOD_ALL.Com_ of 3.02 at SNP rs6139007 (1.613 cM) in the combined sample, a 2-LOD drop support interval at this locus spans 4.8 Mb, 14.2 cM, and 87 RefSeq genes (Figure 3). The combined sample peak SNP is located just 500 bp upstream from the transcription start site of *TRIB3* [GenBank:NM_021158] (0.1-LOD drop from peak), which encodes a regulator of *AKT1* [GenBank:NG_012188] and is expressed mainly in pancreas, bone marrow, and leukocytes [48,49]. The independent sample peak SNP, rs214828 at 8.403 cM, is intronic to gene *TGM3* [GenBank:NM_003245] (0-LOD drop from peak), which encodes a calcium-dependent peptide cross-linking enzyme [50].

While signals at 6q27 and 8q13.2 did not achieve genome-wide significance in either stage of analysis, the consistency of the signal at these loci across the discovery and combined samples suggests these regions may harbor ASD risk variants; each encompasses promising candidate genes, including *SULF1* [GenBank:NM_001128206] (0-LOD drop from peak at 8q13.2, Additional file 2: Figure S1B) located directly under the 8q13.2 linkage peak and whose protein product interacts with growth factors and cytokines in cell signaling [51], and *PARK2* [GenBank:NG_008289] and *RPS6KA2* [GenBank:NM_021135] (1.7- and 0.6-LOD drop, respectively, from peak at 6q27, Additional file 2: Figure S1A), which are both located within rare CNVs identified in ASD cases [52-54].

### Sex-stratified linkage

To identify sex-differential ASD risk loci, we analyzed the multiplex families in two separate groups according to the sex of the affected children in each family: MO and FC (see Methods) [28]. This sex stratification approach has been applied only twice in earlier analyses of exclusively AGRE families, in subgroups only one-third as large as our stage 1 MO and FC subgroups [25,28]. These earlier analyses identified and replicated a genome-wide significant signal at 17q11-q21 in the MO subgroup, which has not been subsequently replicated in larger studies [32]. Using our larger sample, we aimed to identify additional sex-differential risk loci from both the MO and FC subgroups.

Separate non-parametric, genome-wide linkage analyses for ASD affection status in the stage 1 MO (n = 487 families) and FC (n = 314 families) subgroups identified five loci with LOD scores ≥2.2 (Table 2, Figure 2), two of which overlap peaks from all families (6q27, 8p21.2) and three that are suggestive only in either the MO or FC subgroup (1p31.3, 4q26, 8p12).

**Table 2:** Sex subset-specific linkage signals

**Locus**	**Subgroup**	**Stage 1 sample peak LOD**	**Combined sample peak LOD**	**Gene at suggestive linkage peak**	**Sex-differential linkage empirical *P *-value**
1p31.3	MO	2.98	-----	NFIA	**<0.01**
	MO	-----	2.55	NFIA	**<0.0005**
4q26	MO	2.41	1.28	SYNPO2	NS
6q27	MO	2.86	2.07	MLLT4	NS
8p21.2	FC	2.67	1.42	EBF2	**<0.05**
8p12	FC	2.37	1.34	NRG1	**<0.005**

**Bold type** indicates significant sex-differential linkage signals with empirical *P* ≤ 0.05. FC, female-containing; LOD, logarithm of odds; MO, male-only; NS, not significant.

**Figure 5:**
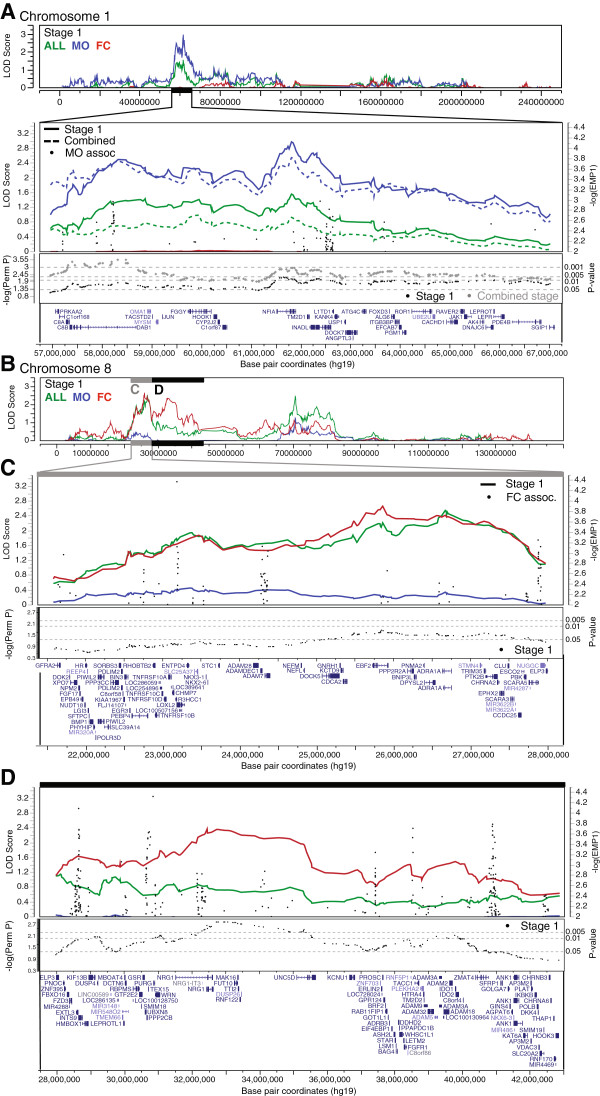
**Significant sex-differential linkage peaks.** Regions of sex subgroup-specific suggestive linkage (logarithm of odds (LOD) ≥2.2) with empirically significant signal enrichment from randomization testing are plotted. **(A)** 1p31.3, MO; top: linkage across full chromosome 1 from all stage 1 family groups; upper-middle: linkage from all stage 1 (solid lines) and combined sample (dashed lines) family groups and association signal from transmission disequilibrium test (TDT; black dots, EMP1 is empirical *P*-value from TDT) in male-only (MO) families across 2-LOD interval from peak LOD; lower-middle: empirical *P*-values from test of linkage subgroup specificity (black = stage 1, gray = combined), dotted lines indicate thresholds corresponding to empirical *P*-values of 0.05, 0.01, and 0.005; bottom: RefSeq gene alignment in 2-LOD interval. **(B)** Linkage across full chromosome 8 from all stage 1 family groups. **(C)** 8p21.2, female-containing (FC), **(D)** 8p12, FC; for each, top: linkage from all stage 1 family groups and TDT association in FC families across 2-LOD interval from peak LOD; middle: empirical *P*-values from stratification permutation tests; bottom, RefSeq gene alignment in 2-LOD interval.

We observed the highest LOD score for the MO subset at chromosome 1p31.3, with LOD_MO.St1_ = 2.98 at rs7521242 (92.905 cM); a 2-LOD interval from this peak SNP spans 10.0 Mb, 13.7 cM, and 48 RefSeq genes. Analysis of this locus in the MO subgroup from the combined sample identified a smaller, but still suggestive, peak LOD_MO.Com_ = 2.55 also at rs7521242 (Figure 5A). In both samples, this MO-specific peak is centered on gene *NFIA* [GenBank:NG_011787] (0-LOD drop from peak), which is expressed in the central nervous system and plays a significant role in glial cell fate determination and in normal development of the corpus callosum [55,56]. Exome sequencing has also identified a *de novo*, non-synonymous, loss of function SNV in an autistic subject in this gene, although the SNV carrier is female [7].

From the FC stage 1 subset, we observed the highest LOD score at chromosome 8p21.2, with LOD_FC.St1_ = 2.67 at rs10111167 (46.967 cM); a 2-LOD interval from this peak SNP spans 6.4 Mb, 9.9 cM, and 73 RefSeq genes (Figure 5C). This FC-specific peak is centered on gene *EBF2* [GenBank:NG_030344] (0-LOD drop from peak), which encodes a transcription factor that may act alongside WNT1 [GenBank:NG_033141] to regulate cellular differentiation during development [57]. However, LOD scores in this region from the combined sample FC subgroup did not reach the suggestive LOD threshold of 2.2. Similarly, the other sex subgroup-specific peaks at chromosomes 6q27 (LOD_MO.St1_ = 2.86), 4q26 (LOD_MO.St1_ = 2.41), and 8p12 (LOD_FC.St1_ = 2.37), only exceeded LOD of 2.2 in the stage 1 sample (Table 1, Table 2).

We next sought to calculate the likelihood of observing the MO and FC signals that surpassed (1p31.3, 4q26, 8p21.2, 8p12) or approached (6q27) the magnitude of the LOD signal from all families by chance. To assess the significance of this signal enrichment in the sex-defined subsets, we performed a randomization test for linkage in 10,000 N-matched subsets of families from the full multiplex family cohort. The empirical *P*-value indicates subset-specific linkage enrichment (empirical *P* < 0.05 at peak SNP) at chromosomes 1p31.3 (MO) in both the stage 1 and the combined samples, and at 8p21.2 (FC), and 8p12 (FC) in the stage 1 sample (Table 2, Figure 5). These results suggest that the stratification of cohorts by proband sex can reveal sex-differential ASD risk loci. However, only the MO locus at 1p31.3 was supported in the combined sample.

Aside from the signal identified by Weiss and colleagues [33] at 20p13 in a non-sex-stratified sample, the only other locus at which genome-wide significant linkage was previously observed was in a MO subset at 17q11 [28]. This signal was subsequently replicated in an additional MO subset of AGRE families [25]. In an attempt to further replicate the signal at this locus, we tested the 407 MO families from the combined stage that were not included in either previous analysis. The most significant linkage *P*-value that we observe within the 2-LOD drop interval from the peak signal observed by Cantor and colleagues [25] (Additional file 3: Table S2) is 0.1003 at rs2014209; thus, we do not independently replicate the MO signal at 17q in this sample.

### Linkage-directed association

We next tested for association with common genotyped and imputed SNPs within the 2-LOD intervals around each linkage peak with LOD ≥2.2 in the stage 1 or the combined sample. For regions with suggestive signals from the MO or FC subsets, association testing was run on the corresponding subset from the combined stage, with the addition of families with multiple affected cousins instead of multiple affected siblings. For regions with suggestive signals from all multiplex families (ALL), association testing was run on all multiplex families from the combined stage with the addition of 508 families with only a single genotyped, affected member. While we observe some clusters of SNPs that approach significance, we did not identify any SNP that survived multiple testing correction for the number of independent SNPs within each region, defined by pairwise LD r^2^ < 0.3. We highlight the top association signals here for the sake of interest, although we emphasize that none pass our correction for multiple comparisons.

The strongest unadjusted association signal within any linkage region occurred in the ALL peak on chromosome 4q13.1 at rs115667468 with a *P*-value of 2.997 × 10^-5^ (after regional correction, *P* = 0.154). This associated SNP is located 7.8 Mb from the SNP at the linkage peak and is intronic to gene *NPFFR2* [GenBank:NM_004885], and the minor allele T was found to be under-transmitted to affected offspring from ALL families (odds ratio = 0.4143). In contrast, the strongest corrected association signal occurred in the FC peak on chromosome 8p21.2 at rs78485638 with a corrected *P*-value of 0.052 (unadjusted *P* = 4.206 × 10^-5^; Figure 5D). This SNP is located 2.7 Mb from the SNP at the linkage peak, is intronic to gene *LOXL2* [GenBank:NG_002318], and the minor allele T was found to be under-transmitted to affected offspring from FC families (odds ratio = 0.4512).

## Discussion

To identify and support genomic loci likely to contain variants contributing to ASD risk in multiplex families from the AGRE collection, we performed linkage analyses in all and sex-stratified subsets of multiplex families followed by targeted association testing in 1,521 families (1,008 multiplex) from the AGRE cohort. The strongest linkage signal that we identified was on chromosome 20p13, which exceeded a LOD score of 3.0 in our combined sample of 1,008 multiplex families. At 20p13, we also replicated in an independent sample from the same AGRE cohort a previous report of significant linkage at this locus [33]. Analyses of sex-defined family subgroups and randomization testing for signals from these subgroups identified a locus at chromosome 1p31.3 that showed significant linkage in the MO subgroup in both the stage 1 and combined samples, and loci at chromosomes 8p21.2 and 8p12 that showed significant linkage in the FC subgroup only in the stage 1 sample. No genotyped or imputed common SNPs within any linked region proved to be significantly associated with ASD, an observation consistent with a model where the influence of multiple loci of very small effect size, or of rare variants, contributes to ASD.

The linkage signal at 20p13 is especially noteworthy as it was the most significant signal that we observed in our combined sample with a LOD_All.Com_ of 3.02, and it also meets the criterion for an independent replication of genome-wide significant linkage [43]. In addition, as both this study and the study by Weiss and colleagues [33] were conducted using the AGRE collection, a sample that has been uniformly ascertained and evaluated over time, this is a clear replication. A 2-LOD interval from the combined sample peak spans such potential risk candidate genes as *NRSN2* [GenBank:NM_024958] (0.3-LOD drop from peak), a gene expressed throughout the cerebral cortex, thalamus, hypothalamus, and in Purkinje cells [58], and *CSNK2A1* [GenBank:NG_011970] (0.5-LOD drop from peak), which encodes a protein involved in the regulation of circadian rhythms [59].

For nearly all linked regions identified in the discovery samples, including 20p13, LOD scores decreased when analyzed in the combined sample despite the increased sample size, consistent with previous observations of genetic heterogeneity [60-62]. However, it is interesting to note that those signals that were confirmed or replicated in the combined or independent sample were initially observed by tests of relatively large discovery samples. This is because, like association testing, a very large number of small pedigrees are critical to identify robust linkage signals [63]. In smaller samples, signal fluctuations between analyses are caused by extreme heterogeneity of genetic risk loci for ASD [60,61,64], such that each analysis identifies different, but potentially true, risk loci. Since linkage peaks represent deviations from expected proportions of affected family members sharing two, one, or zero alleles identical by descent at a particular locus, linkage analyses for complex traits are particularly sensitive to the composition of families included in any one test. If, as projected, there are close to 1,000 risk genes for ASD [12], then the chances that a sufficient proportion of families in current sample sizes share the same risk locus are low.

We recognize that the power we have to detect linkage in our analyses is for complete linkage with loci of modest genetic effects on ASD risk [63], and so it is not surprising that we do not observe genome-wide significant linkage in either the stage 1 or extended, combined stage samples, and that most suggestive signals fail to increase in the extended sample. However, we emphasize that only now are we approaching the sample sizes necessary to detect significant linkage in the face of locus heterogeneity in cohorts comprised of small pedigrees, and that any possible reduction of locus heterogeneity (for example, by testing families ascertained by the same cohort as is done here), will be key to identify replicable linkage signals.

Rational stratification of cohorts into subgroups based on a shared trait may also facilitate the discovery of risk loci by increasing the relative homogeneity of specific genetic risk factor(s) in the subgroup. Here, we stratified our sample by the sex of the probands within each family to identify loci with sex-differential relationships with ASD risk, and found significant risk loci at chromosome 1p31.3 (MO), 8p21.2 (FC), and 8p12 (FC). At 1p31.3 and 8p12, linkage signals from the MO and FC subsets, respectively, were significantly stronger than the signal from the full, non-stratified cohort, suggesting that this sex-based stratification approach can indeed reduce the genetic heterogeneity within each subgroup that would otherwise obscure signals at these loci.

We note that these loci should not be interpreted as simply sex-specific. Although we cannot say that the MO group is perfectly restricted to families who carry solely male-specific variants and therefore only have, and would ever have, affected male children, we do assume that the MO subgroup is substantially enriched for families who carry risk variants that are more penetrant in males. Thus, signals specific to the MO subset are more likely to be male-specific. In contrast, since the FC group includes affected brothers of autistic girls, FC signals are best interpreted not as female-specific, but as female-affecting risk loci, in accord with a hypothesis that only a subset of ASD risk loci are penetrant in females [7,9-13,65].

The significant MO and FC signals identified here implicate regions containing promising candidate genes that warrant further exploration by targeted re-sequencing (for example, [66-69]). The MO peak at 1p31.3 is located directly over *NFIA* [GenBank:NG_011787], whose gene product has transcription factor activity and has been implicated in central nervous system development [55,56]. Rare deletions encompassing this gene have been identified in subjects with ASD [70], as well as *de novo* mutations [7]. The FC peak at 8p21.2 spans several candidates, including *STC1* [GenBank:NG_029711] (1.1-LOD drop from peak), which encodes a glycoprotein regulated by calcium that may act to protect neurons from ischemia and hypoxia [71], and neurofilament genes *NEFM* [GenBank:NG_008388] and *NEFL* [GenBank:NG_008492] (both 1.1-LOD drop from peak) whose products likely function in transport to neuronal projections [72]. Potentially relevant to sex-differential risk, *GNRH1* [GenBank:NG_016457] (0.7-LOD drop from peak) is also located within this linkage region, and mutations in this gene are likely to affect gonadal function [73], perhaps differentially modulating downstream manifestation of ASD risk factors in males and females. The neighboring FC peak at chromosome 8p12 is located directly over *NRG1* [GenBank:NG_012005] (0-LOD drop from peak), a known schizophrenia risk gene [74,75].

A similar stratification approach for the identification of male-specific and female-affecting ASD risk loci has been successfully applied previously by Stone and colleagues [28] to an early iteration of the AGRE cohort, as well as by Szatmari and colleagues [32] to a sample that included a subset of AGRE families, and Lamb and colleagues [30] and Schellenberg and colleagues [29] to other ASD family samples. No convergence in linkage signals was observed across these studies. We predicted that our ability to identify novel, sex-differential ASD risk loci in the present study would be aided by a greater than three-fold increase in subjects exclusively from AGRE as compared to those used by Stone and colleagues [28] and by the increase in coverage afforded by dense SNP data in lieu of several hundred microsatellite markers. However, the results from our sex-stratified analyses do not reach genome-wide significance and also do not align with findings from earlier sex stratification analyses, including Stone and colleagues [28] who reported a linkage peak at 17q11-q21 in MO families that was subsequently replicated [25]. This variability between studies is again consistent with extreme risk locus heterogeneity in ASD [76,77], with each analysis of a different combination of ASD families identifying different linked regions.

Under the assumption of genetic heterogeneity, for complex traits such as ASD, it is possible that the various loci identified by analyses of different family sets flag true sites for ASD risk in a proportion of the families tested. To pursue this, linked loci will need to be investigated more closely to identify the precise variants that effectively increase ASD risk. This has so far proven challenging, as association testing of densely mapped common SNPs within linkage peaks has failed to definitively identify risk variants, both in the present study and in previous work [33].

Although our analyses are likely underpowered to identify regionally significant associations with common variants of small effect size, it is alternatively possible that rare variants, not explicitly tested here, contribute to the heritable component of ASD risk. For example, rare variants, shared between siblings but private to each nuclear family, may cluster in the same gene or set of genes. Since rare variants are less likely to be tagged by common SNPs, they should be more readily localized by allele-agnostic linkage analyses than association testing. As in gene discovery studies of sporadic ASD cases, sequencing of functional genomic features in linked regions will be necessary to identify rare variants and evaluate their role in familial ASD risk. In either case, larger family-based cohorts will be needed to improve power.

## Conclusions

We conclude that the use of linkage analyses in multiplex family cohorts has complementary utility to genome-wide association studies for the investigation of the familial, inherited contribution to ASD risk. This is especially the case in the context of rare variants in human disease (for example, [78,79]). Additionally, the use of a sex stratification approach facilitates the identification of risk loci that are differentially associated with ASD in families with autistic sons versus daughters. However, further work is needed to determine which gene(s) or genetic features within linked regions, especially at chromosome 20p13, replicated here, harbor the variants responsible for increasing familial and sex-differential genetic risk for ASD. Exploring this in detail via targeted sequencing in large cohorts will be necessary to elucidate the common versus rare genetic contributions to ASD.

## Abbreviations

AGRE: Autism Genetics Resource Exchange; ALL: all families; ASD: autism spectrum disorders; CNV: copy number variant; FC: female-containing; IL: interleukin; LOD: logarithm of odds; MO: male-only; SNP: single nucleotide polymorphism; SNV: single nucleotide variant; TDT: transmission disequilibrium test.

## Competing interests

The authors declare that they have no competing interests.

## Authors’ contributions

DMW carried out the linkage and association studies and drafted the manuscript. JKL performed preprocessing and quality control on genotype data and provided assistance with genetic analyses. RL carried out the genotype imputation for all samples. RMC advised on the design and interpretation of the study and helped to draft the manuscript. DHG conceived of the study, participated in its design and interpretation, and helped to draft the manuscript. All authors read and approved the final manuscript.

## Supplementary Material

Additional file 1: Table S1 Genotyped families and cases from Autism Genetics Resource Exchange (AGRE). *Additional members from families partially genotyped at earlier stage. Click here for file

Additional file 2: Figure S1 Linkage observed at suggestive threshold in both stage 1 and combined samples. Regions of suggestive linkage (logarithm of odds (LOD) >2.2) observed in both the full sample (ALL) from stage 1 and combined stage analyses. A) 6q27, B) 8q13.2-3; for each, top: linkage across full chromosome from all stage 1 family groups; middle: linkage from stage 1 (solid line) and combined samples (dashed line) and association signal from transmission disequilibrium test (TDT) in all combined stage families (black dots, EMP1 is empirical *P*-value from TDT) across 2-LOD interval from peak LOD; bottom: RefSeq gene alignment in 2-LOD interval. Click here for file

Additional file 3: Table S2 Peaks and boundaries for linkage regions identified in discovery samples. The location of the peak logarithm of odds (LOD) score by chromosomal band, single nucleotide polymorphism (SNP) or microsatellite, hg19 base pair coordinate(s), and genetic position (cM) is reported on the left side of the table. The left and right boundaries by SNP, hg19 base pair coordinate, and genetic position of a 2-LOD drop interval from the peak marker are reported in the center and right sections of the table, respectively. Click here for file
