# Supplementary material for: Replication of linkage at chromosome 20p13 and identification of suggestive sex-differential risk loci for autism spectrum disorder
Source: Mol Autism. 2014 Feb 17;5:13. doi: 10.1186/2040-2392-5-13 (PMC3942516; doi:10.1186/2040-2392-5-13)
Supplement: Additional file 1: Table S1 — Genotyped families and cases from Autism Genetics Resource Exchange (AGRE). *Additional members from families partially genotyped at earlier stage. [file 2040-2392-5-13-S1.doc]

**Additional file 1: Table S1. Genotyped families and cases from AGRE**

|  | **Stage** | **Platform** |  | **Total** | **Multiplex** | **MO** | **FC** |
| --- | --- | --- | --- | --- | --- | --- | --- |
| Combined sample | Stage 1 | Illumina 550K | Families | 941 | 704 | 431 | 273 |
|  |  |  | Cases (females) | 1729 (368) | 1481 (319) | 886 (0) | 595 (319) |
|  |  | Illumina Omni-1 | Families | 250+29* | 128 | 77 | 51 |
|  |  |  | Cases (females) | 381 (78) | 232 (52) | 134 (0) | 98 (52) |
|  | Stage 2 | Illumina Omni-1 | Families | 116+11* | 27 | 9 | 18 |
|  |  |  | Cases (females) | 141 (39) | 51 (20) | 19 (0) | 32 (20) |
|  |  | Illumina Omni-2.5 | Families | 280+3* | 151 | 89 | 62 |
|  |  |  | Cases (females) | 478 (102) | 347 (82) | 188 (0) | 159 (82) |

Additional file 1: Table S1 legend:

*Additional members from families partially genotyped at earlier stage
