# Supplementary figures and images for: Replication of linkage at chromosome 20p13 and identification of suggestive sex-differential risk loci for autism spectrum disorder
Source: Mol Autism. 2014 Feb 17;5:13. doi: 10.1186/2040-2392-5-13 (PMC3942516; doi:10.1186/2040-2392-5-13)

## A Chromosome 6

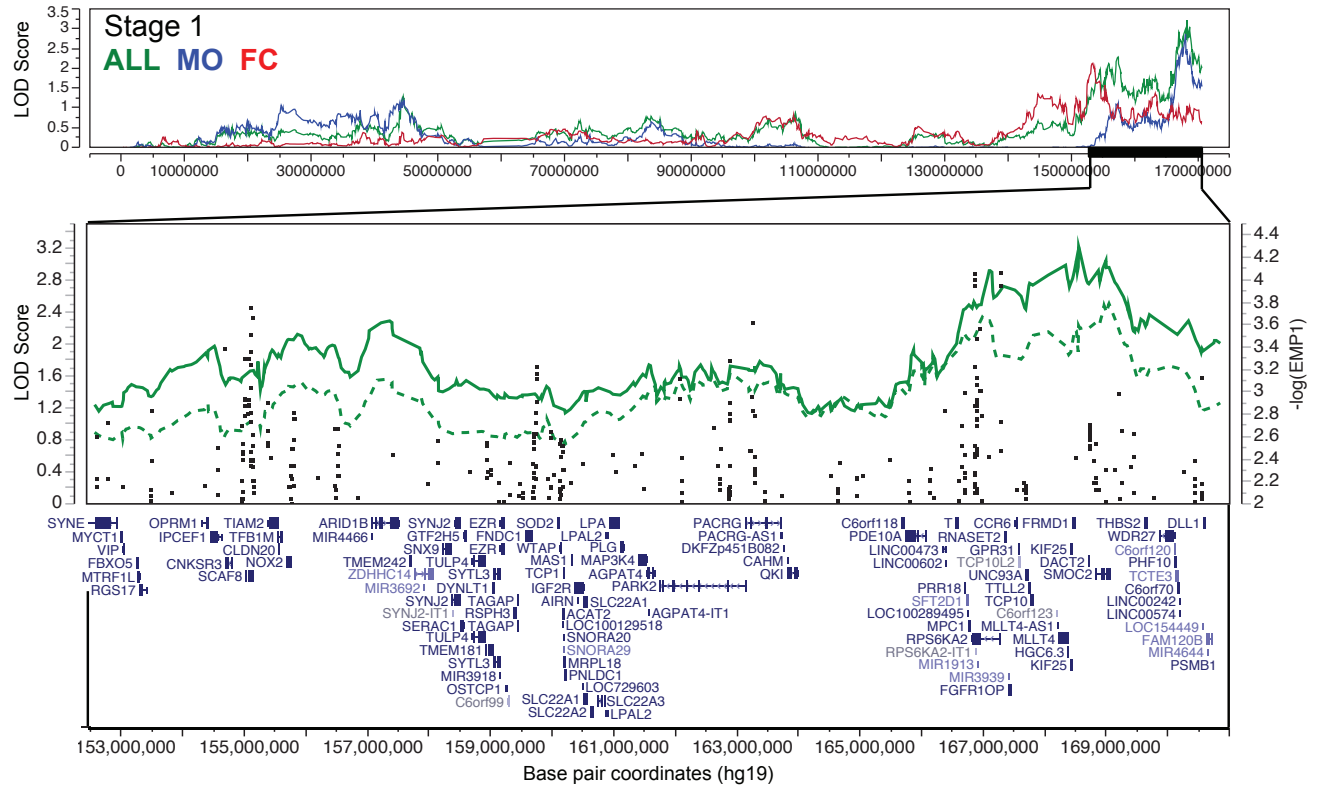

## B Chromosome 8

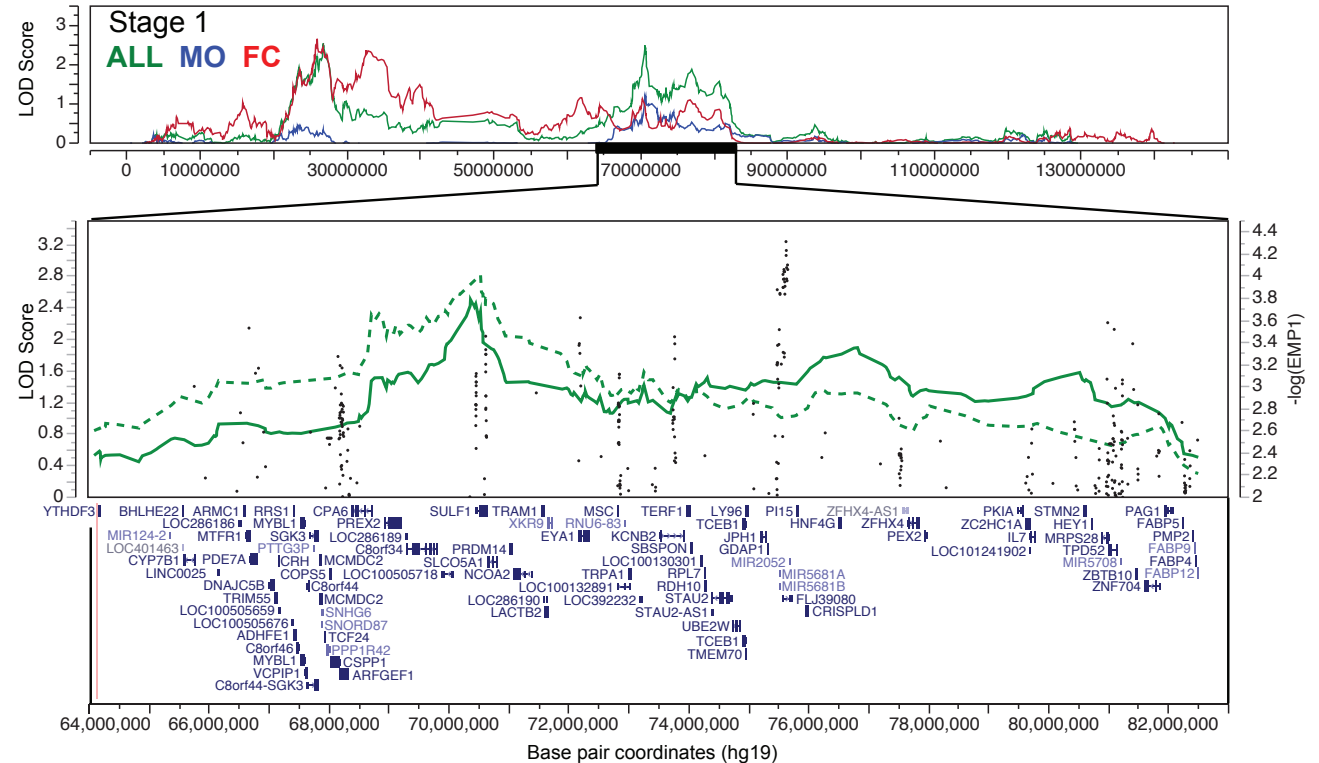

Supplement: Additional file 2: Figure S1 — Linkage observed at suggestive threshold in both stage 1 and combined samples. Regions of suggestive linkage (logarithm of odds (LOD) >2.2) observed in both the full sample (ALL) from stage 1 and combined stage analyses. A) 6q27, B) 8q13.2-3; for each, top: linkage across full chromosome from all stage 1 family groups; middle: linkage from stage 1 (solid line) and combined samples (dashed line) and association signal from transmission disequilibrium test (TDT) in all combined stage families (black dots, EMP1 is empirical P-value from TDT) across 2-LOD interval from peak LOD; bottom: RefSeq gene alignment in 2-LOD interval. [file 2040-2392-5-13-S2.pdf]
