# Supplementary material for: Replication of linkage at chromosome 20p13 and identification of suggestive sex-differential risk loci for autism spectrum disorder
Source: Mol Autism. 2014 Feb 17;5:13. doi: 10.1186/2040-2392-5-13 (PMC3942516; doi:10.1186/2040-2392-5-13)
Supplement: Additional file 3: Table S2 — Peaks and boundaries for linkage regions identified in discovery samples. The location of the peak logarithm of odds (LOD) score by chromosomal band, single nucleotide polymorphism (SNP) or microsatellite, hg19 base pair coordinate(s), and genetic position (cM) is reported on the left side of the table. The left and right boundaries by SNP, hg19 base pair coordinate, and genetic position of a 2-LOD drop interval from the peak marker are reported in the center and right sections of the table, respectively. [file 2040-2392-5-13-S3.doc]

**Additional file 3: Table S**2. Peaks and boundaries for linkage regions identified in discovery samples

|  |  | **Peak LOD** | | | | **Left boundary** | | | **Right boundary** | | |
| --- | --- | --- | --- | --- | --- | --- | --- | --- | --- | --- | --- |
| **Source** | **Sample group** | **Locus** | **Peak marker (source)** | **Peak base pair (hg19)** | **Peak cM in combined sample** | **SNP** | **Physical position (bp)** | **Genetic position (cM)** | **SNP** | **Physical position (bp)** | **Genetic position (cM)** |
| Liu et al. (2001) | ALL | 5p13.1 | D5S2494 | 40,218,211- 40,218,452 | 64.64 | rs10042912 | 24,902,336 | 44.64 | rs1108159 | 61,556,730 | 76.64 |
|  | ALL | Xq26.1 | DXS1047 | 129,075,297- 129,075,586 | 131.58 | rs743821 | 88,348,549 | 90.00 | rs1128863 | 154,356,862 | 189.93 |
| Yonan et al. (2003) | ALL | 5p13.1 | D5S2494 | 40,218,211- 40,218,452 | 64.64 | rs10042912 | 24,902,336 | 44.64 | rs10040902 | 55,896,630 | 71.64 |
|  | ALL | 11p13-11.2 | D11S1392 & D11S1993 | 34,640,296- 43,609,920 | 55.19 | rs2945096 | 28,873,413 | 45.19 | rs4326823 | 69,173,889 | 75.19 |
|  | ALL | 17q11.2 | D17S1800 | 29,936,783- 29,937,104 | 61.51 | rs1382779 | 20,527,718 | 49.51 | rs2278868 | 46,262,171 | 77.51 |
| Stone et al. (2004) | MO | 17q11 | D17S1294 & D17S798 | 28,382,520- 31,289,812 | 61.18 | rs11868474 | 27,659,431 | 55.18 | rs11080254 | 32,479,505 | 65.18 |
|  | FC | 4q32.3-35.1 | Not provided | Unknown | 160.00 | rs13133181 | 143,610,293 | 140.00 | rs7665469 | 174,229,762 | 168.00 |
| Cantor et al. (2005) | MO | 17q21 | D17S1299 | 38,994,307- 38,994,621 | 71.84 | rs2191090 | 26,828,497 | 53.84 | rs1492233 | 50,579,289 | 80.84 |
| McCauley et al. (2005) | ALL | 3p25.3 | D3S3691 | 8,840,396- 8,840,778 | 25.23 | rs7615939 | 2,832,318 | 7.23 | rs6804427 | 19,683,373 | 40.23 |
| Szatmari et al. (2007) | MO | 5q12 | rs673743 | 62,867,084 | 77.03 | rs679895 | 29,091,685 | 47.987 | rs4572979 | 77,606,215 | 93.003 |
|  | MO | 9q33.3 | rs536861 | 128,313,444 | 133.88 | rs16919567 | 103,624,177 | 104.524 | rs2606357 | 141,011,581 | 160.792 |
|  | ALL | 11p12 | rs2421826 | 35,230,605 | 51.81 | rs11032242 | 33,390,547 | 48.332 | rs7925914 | 59,708,244 | 64.955 |
|  | FC | 11p12 | rs1039205 | 36,711,834 | 55.58 | rs7933966 | 32,875,597 | 47.796 | rs2902421 | 45,016,421 | 62.831 |
| Weiss et al. (2009) | ALL | 6q27 | rs4708679 | 168,573,159 | 186.30 | rs2695253 | 152,926,517 | 158.82 | rs12205313 | 170,851,436 | 189.82 |
|  | ALL | 20p13 | rs723477 | 289,362 | 1.20 | rs722829 | 169,701 | 0.43 | rs6047800 | 2,252,739 | 8.20 |
| Current study  Stage 1 sample | MO | 1p31.3 | rs7521242 | 61,803,889 | 87.57 | rs4628 | 56,961,756 | 79.69 | rs4655616 | 67,002,765 | 93.42 |
|  | ALL | 4q13.1 | rs2128887 | 65,108,929 | 77.63 | rs7693131 | 42,652,004 | 63.94 | rs10518234 | 81,152,247 | 88.45 |
|  | MO | 4q26 | rs6828669 | 119,947,135 | 121.90 | rs2765 | 104,510,766 | 107.85 | rs6535038 | 135,643,583 | 131.60 |
|  | ALL | 6q27 | rs4708676 | 168,555,227 | 186.32 | rs9371581 | 152,629,586 | 158.26 | rs12205313 | 170,851,436 | 189.82 |
|  | MO | 6q27 | rs1132306 | 168,343,838 | 185.95 | rs1584535 | 156,377,582 | 164.81 | rs12205313 | 170,851,436 | 189.82 |
|  | FC | 8p21.2 | rs10111167 | 25,863,992 | 44.42 | rs6587004 | 21,602,192 | 37.60 | rs4732823 | 27,973,215 | 47.52 |
|  | ALL | 8p21.2 | rs13257637 | 26,673,902 | 46.04 | rs1120415 | 20,894,128 | 35.98 | rs2670683 | 29,272,247 | 49.61 |
|  | FC | 8p12 | rs2466062 | 32,443,090 | 53.28 | rs4732823 | 27,973,215 | 47.52 | rs4608108 | 48,515,523 | 62.13 |
|  | ALL | 8q13.2 | rs2244817 | 70,376,825 | 79.80 | rs4739066 | 64,104,092 | 75.18 | rs2581529 | 82,503,999 | 92.76 |
| Current study  Combined sample | MO | 1p31.3 | rs7521242 | 61,803,889 | 87.57 | rs17105974 | 50,620,945 | 72.12 | rs17129664 | 67,583,298 | 94.42 |
|  | ALL | 6q27 | rs6931082 | 169,047,090 | 187.18 | rs2473609 | 151,626,671 | 155.72 | rs12205313 | 170,851,436 | 189.82 |
|  | ALL | 8q13.2 | rs4738003 | 70,542,552 | 79.92 | rs6472077 | 64,028,399 | 75.13 | rs7840008 | 81,058,344 | 90.05 |
|  | ALL | 20p13 | rs6139007 | 360,789 | 1.61 | rs6139074 | 63,244 | 0.28 | rs2423067 | 4,817,968 | 14.48 |

Additional file 3: Table S2 legend:

The location of the peak LOD score by chromosomal band, SNP or microsatellite, hg19 base pair coordinate(s), and genetic position (cM) is reported on the left side of the table. The left and right boundaries by SNP, hg19 base pair coordinate, and genetic position of a 2-LOD drop interval from the peak marker are reported in the center and right sections of the table, respectively.
